# Methods and experiences of a collaborative research project carried out by academic clinical researchers and experts by experience

**DOI:** 10.1192/j.eurpsy.2024.1498

**Published:** 2024-08-27

**Authors:** M. Ameel, K. Hirsma, T. Majalahti, P. Soininen

**Affiliations:** ^1^Psychiatry, Helsinki University Hospital; ^2^Psychiatry, University of Helsinki, Helsinki; ^3^Nursing Science, University of Turku, Turku; ^4^ Helsinki University Hospital; ^5^University of Helsinki, Helsinki, Finland

## Abstract

**Introduction:**

Patient or service user participation in research and development is seen as essential in health research, including in topics within psychiatry. The process and depth of research collaboration can vary and is not always described adequately.

**Objectives:**

The objective is to describe the collaborative methods and the experiences of experts by experience and academic researchers in a research project on patients’ experiences of remote care in psychiatric settings during and after the COVID-19 pandemic.

**Methods:**

We describe our collaborative methods and experiences using the INVOLVE key features (www.involve.nihr.ac.uk).

**Results:**

Collaboration started with an open discussion on research aims and role definitions. Collaborative methods included teaching and training sessions on interview methodologies, collaboratively writing and evaluating documents for ethical approval and research permission, collaboratively planning the recruitment process, preparation, and conducting research interviews and analysis. On-line and in-person meetings have been essential for an an-going dialogue and reflection. The methods and experiences are described in more detail in Table 1.

Openness and building trust have been important and time was needed to achieve these. All academic researchers had been actively working with experts by experience in the clinical settings before the research project. The collaboration in the current study has emphasized the need for active involvement of experts with experience throughout the research process. For the experts by experience, the project has provided new insight into academic research and given them confidence in their ability to participate meaningfully in a collaborative study project. The academic researchers valued the sense of significance of the research topic and shared decision-making that the collaboration has brought into the project.

**Image:**

**Figure:**
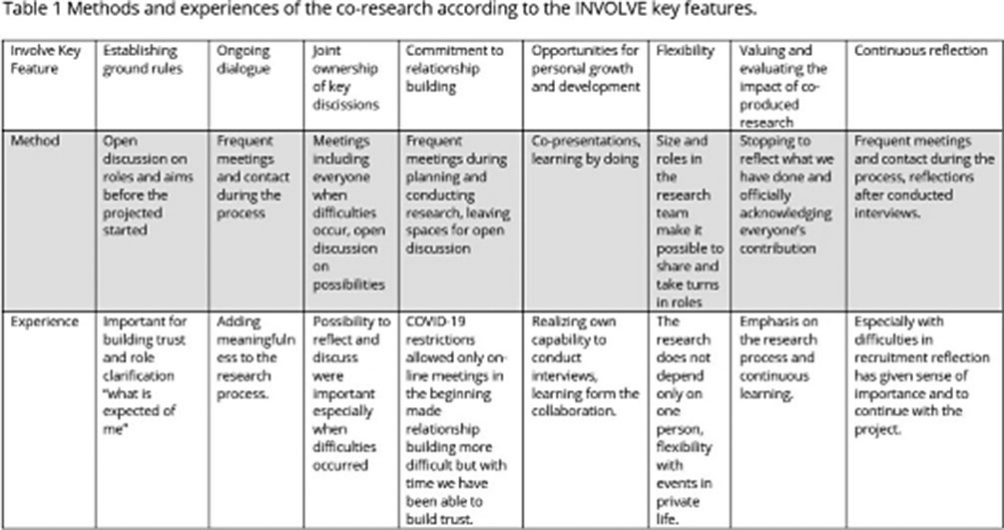

**Conclusions:**

Collaborative research needs time to build trust and to clearly define the roles of participants, from the opening stage of the process. Continuous learning during the research process is emphasized. Since different research methodologies arise from various theoretical backgrounds, we suggest adding a topic on research theory to the INVOLVE key features.

**Disclosure of Interest:**

None Declared

